# Comparison of Standard 7-Field, Clarus, and Optos Ultrawidefield Imaging Systems for Diabetic Retinopathy (COCO Study)

**DOI:** 10.1016/j.xops.2023.100427

**Published:** 2023-11-11

**Authors:** Nicole Duncan, Nancy Barrett, Kathleen Schildroth, Jonathan S. Chang, Roomasa Channa, Kelsey Rickels, Amitha Domalpally, Barbara Blodi

**Affiliations:** 1 Department of Ophthalmology and Visual Sciences, University of Wisconsin School of Medicine and Public Health, Madison, Wisconsin; 2 McPherson Eye Research Institute, University of Wisconsin-Madison, Madison, Wisconsin

**Keywords:** 7-Field imaging, Diabetic retinopathy, ETDRS scale, Retinal imaging, Ultra-widefield imaging

## Abstract

**Purpose:**

The purpose of this study was to compare diabetic retinopathy (DR) severity levels assessed from 7 standard-field stereoscopic color photographs on a 35° fundus camera to both Clarus and Optos ultrawidefield color images.

**Design:**

Cross-sectional, comparative imaging study.

**Participants:**

Participants with DR imaged at a single-center retina practice.

**Methods:**

Participants were imaged on 3 cameras at a single visit with the Topcon 35° fundus camera, Clarus, and Optos. The DR Severity Scale (DRSS) level was determined within the 7-field (7F) area of each image set using the ETDRS scale. An additional global DRSS was assigned for both Clarus and Optos images using the entire visible retina. Weighted kappa (wκ) measured the agreement between cameras.

**Main Outcome Measures:**

The primary outcome was a 3-way comparison of DRSS level within the 7F area imaged on the 3 cameras. Secondary outcomes included a comparison of the DRSS obtained with standard 7F imaging to the global DRSS of Clarus and Optos and a comparison of the global DRSS between Clarus and Optos only.

**Results:**

Ninety-seven eyes (50 participants) were evaluated. Agreement within 1-step of ETDRS levels between standard 7F imaging and Clarus 7F was 90.1% (wκ = 0.65), and with Optos 7F in 85.9%, (wκ = 0.58). Agreement within 1-step between standard 7F imaging and Clarus global was 88.9% of eyes (wκ = 0.63), and Optos global was 85.7%, (wκ = 0.54). Agreement between Clarus and Optos global DR level within 1-step was 89.1% (wκ = 0.68). Intergrader agreement for the 7F ETDRS level was 96% for standard 7F imaging, 98% for Clarus, and 95.5% for Optos.

**Conclusions:**

These findings suggest that when evaluating the 7F area on Clarus and Optos, DR severity grades are comparable to standard 7F imaging. However, it is important to understand the unique attributes and differences of each fundus camera when changing the type of system used in a clinical setting due to upgrading equipment. Additionally, if the facility has access to > 1 device, there should not be an exchange between cameras for the same patient.

**Financial Disclosure(s):**

Proprietary or commercial disclosure may be found in the Footnotes and Disclosures at the end of this article.

Retinal imaging has been used for almost a century as a means of documenting retinal pathology.^1^ Fundus cameras utilize white light photography to capture a 2-dimensional image of the posterior part of the eye, allowing direct visualization of the retina. A standard camera can acquire 30 to 50° of true color images (red, blue, and green channels) for imaging the optic nerve and macula. To visualize additional regions of the retina, the photographer must map out additional fields in the midperipheral retina. The Diabetic Retinopathy Study described image acquisition protocols to capture 7 fields (2 posterior and 5 midperipheral) of the retina to establish the region of interest for the assessment of diabetic retinopathy (DR).^2^ For the last 30 years, the 7-field (7F) imaging protocol has been the gold standard for DR clinical trials.^3^, ^4^, ^5^

Ultrawidefield color (UWF-C) cameras can image almost 200° or 80% of the retina in a single capture. The ultrawidefield (UWF) images include the 7 fields of the posterior and midperipheral retina (comparable in area to standard photography) along with the far peripheral retina.^6^ Among the various UWF cameras available, Optos (Optos PLC) is the most studied for the assessment of DR and has improved our overall understanding of the disease.^7^, ^8^, ^9^, ^10^, ^11^, ^12^ The Optos UWF imaging system can acquire 200° retinal images, called Optomap, using confocal technology to allow assessment of the retina in 1 image.^13^ Multiple studies have compared Optos UWF imaging to standard 7F retinal photography for assessment of DR and found the 2 comparable.^6^^,^^11^^,^^14^

In 2017, Zeiss (Carl Zeiss Meditec AG) introduced the Clarus system, adding to the list of commercially available UWF imaging systems. Clarus utilizes both confocal imaging and true color photography to capture 133° of the retina. A montage of 2 Clarus images can provide a field of view equivalent to that from the Optos camera.^15^

The field of retinal imaging has witnessed significant advancements in recent years, resulting in improved reliability and accuracy of diagnosis. Ultrawide image capture has emerged as a convenient and user-friendly technique that benefits both patients and technicians. There is a need to validate the ETDRS scale for DR severity, developed for standard 7F photography, in UWF systems. In this investigation, the COCO study aims to determine whether DR assessment using the ETDRS scale with UWF imaging is comparable with standard 7F imaging. A second goal is to compare DR assessment between Clarus and Optos UWF imaging. Establishing the comparability of all 3 cameras would represent a substantial improvement over existing imaging technology and expand the availability of UWF imaging machines to clinical trials.

## Methods

### Study Design

This cross-sectional, single-center, comparative imaging study was performed after obtaining approval from the University of Wisconsin’s Institutional Review Board. The investigation was conducted following the tenets of the 1964 Declaration of Helsinki. All clinical information was acquired and secured in conformity with the regulations of the Health Insurance Portability and Accountability Act. Throughout the recruitment process, participants were stratified by the treating physician with the aim of ≥ 15 eyes within each DR category using the International Clinical Diabetic Retinopathy severity scale: mild, moderate, and severe non-proliferative DR (NPDR) levels and proliferative DR (PDR).^16^ The International Clinical Diabetic Retinopathy scale was employed for stratification and a preliminary assessment because of its ease of use in a clinical setting. A retina specialist obtained written informed consent from each participant before participation in the study.

### Patients

The study population was patients aged 18 to 90 years old with either type 1 or type 2 diabetes who presented at a single center of a multiphysician retina practice. Patients were excluded if they had a history of panretinal laser photocoagulation or vitrectomy, or if they were unable to tolerate ophthalmic imaging. Additional exclusion criteria included ocular media of insufficient clarity to obtain acceptable UWF images and the presence of confounding abnormalities, such as age-related macular degeneration or retinal vein occlusion. A diagnosis of macular edema or treatment with anti-VEGF was not an exclusion criterion.

### Imaging Protocol

**Figure 1:**
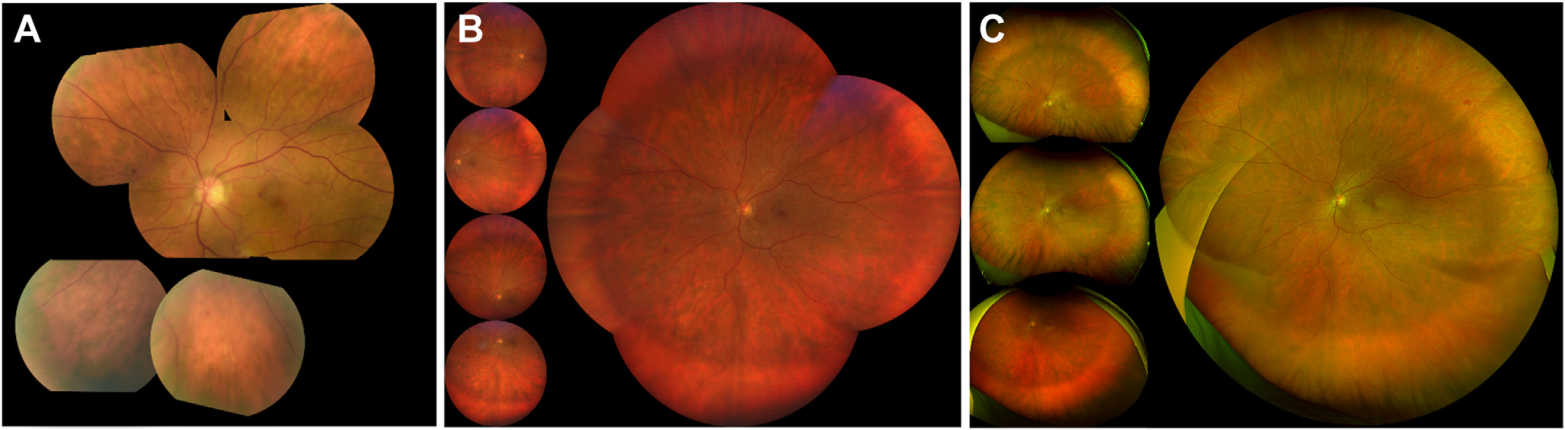
Montages from each modality. Standard 7-field imaging (**A**) shows the standard 7 fields. Clarus ultrawidefield imaging (**B**) shows the individual nasal, temporal, superior, and inferior images next to the montage. Optos ultrawidefield imaging (**C**) shows the individual superior, on-axis, and inferior images next to the montage.

Participants underwent pupillary dilation and consecutive imaging of each eligible eye with standard 7F imaging, Optos, and Clarus cameras, in that order. All images were captured at a single visit by a certified ophthalmic photographer in the Clinical Eye Research Unit using standard image acquisition protocols (Fig 1).

#### Standard 7F Imaging Protocol

The modified 7-standard stereoscopic fields were imaged in color at 35° magnification using Topcon 50DX. This procedure abided by the ETDRS 7-standard field protocol except in the position of 2 fields: field 1M and field 3M were both modified to include the center of the macula. Overall, there were 3 fields depicting the optic disc and macula and 4 fields showing the more peripheral nasal and temporal retina.

#### Clarus UWF Imaging Protocol

Images were acquired using the Zeiss Clarus system (Clarus 500 and 700: version 1.0.2 or higher). The central field was captured at 133° in stereoscopic pairs. Four additional photographs were taken to capture the temporal, superior, nasal, and inferior fields. The 4 photographs were montaged into 1 image to provide a 267° field of view.

#### Optos UWF Imaging Protocol

Images were taken using the Optos Color UWF system (Vantage 3.4, Advance 4.2, or higher). Two on-axis 200° color photographs were captured stereoscopically. Additionally, 1 superior 200° and 1 inferior 200° images were obtained. Lastly, the automontage feature combined and blended individual photographs to produce a single image with a maximum field of view. In both protocols, care was taken to minimize imaging adjacent lids and lashes such that < 10° of the field of view was compromised in any picture.

### Grading Protocol

All retinal images were evaluated by 4 certified graders at the Wisconsin Reading Center using calibrated monitors. Each image set was independently evaluated by 2 readers. In the case of a discrepancy between the 2 readers of ≥ 1 steps on the Diabetic Retinopathy Severity Scale (DRSS), a senior grader provided adjudication. The adjudicator’s evaluation was recorded as the final grade. A washout period of ≥ 2 days occurred between evaluating standard 7F imaging, Clarus UWF-C, and Optos UWF-C images for the same eye.

#### Standard 7F Grading Protocol

The DRSS for each eye was assessed according to the ETDRS scale. This sequence stratifies DR severity into 16 alphanumeric categories, which were then recoded to a linear ETDRS 12-step value. Graders evaluated paired color images using a handheld stereoscopic viewer on a color-calibrated monitor. After reviewing all fields, a grade was assigned based on the most severe DR lesions seen in the eye per the ETDRS grading protocol.^17^ If the macula or optic disc could not be evaluated, or ≥ 2 peripheral fields were compromised by ≥ 50%, the image set was deemed ungradable. Reasons for poor image quality were assessed and documented as patient-related, technical, or both. Proliferative DRSS levels were assigned when appropriate lesions were seen, regardless of image quality.

#### Clarus and Optos UWF Grading Protocols

Clarus images were evaluated in Zeiss Clarus Review software. A grid was automatically placed to outline the ETDRS 7 fields. The periphery was not masked. Optos images were uploaded to custom-built software (Excelsior software; https://meritcro.com/software/) for evaluation. Graders manually placed the ETDRS 7F grid on each image and masked the far-peripheral retina.

An additional global DR severity level was assigned for each UWF-C image set using the entire visible retina based on previously published global DRSS.^14^ With Clarus and Optos images, graders added in the peripheral retina from each of the peripheral fields and evaluated the total retina with the same criteria used within the standard 7F imaging; in the UWF images, the DRSS global level could worsen if the additional peripheral lesions warranted a change. When grading the global DRSS, ≥ 4 of the 5 far-peripheral fields must be present and of good quality. If ≥ 2 fields were compromised by ≥ 50%, the global DRSS assigned was ungradable.

### Statistical Analysis

The sample size calculation was based on the κ agreement between standard 7F and UWF-C devices for ETDRS severity levels. Based on the prediction that one-half of the participants would have both eyes meeting inclusion criteria and assuming 20% data loss due to image quality, a sample size of 60 participants with approximately 70 eyes was calculated.

ETDRS level agreement was cross-tabulated and κ statistics were calculated and assessed based on Landis and Koch: < 0.20, poor; 0.21 to 0.40, fair; 0.41 to 0.60, moderate; 0.61 to 0.80, substantial; and 0.81 to 1.00, almost perfect strength of agreement.^18^ Unweighted κ was used to avoid potential bias by weighing. The agreement was also tested for each severity level and κ statistics were analyzed. Agreement is assessed as exact, within 1 step and within 2 steps. Exact agreement means that the grading was identical on both cameras, e.g., ETDRS level was 5 for both cameras. Within 1-step implies that the grading can differ by 1 point, e.g., if image from 1 camera was graded as 5, the other camera image was graded as 4, 5, or 6. Within 2-step agreement broadens this range, allowing for a difference of up to 2 points, e.g., in the above scenario of step 5 on 1 camera, the other camera could have a range of 3 to 7. Agreement within 1-step of the ETDRS scale is presented in the results because this standard represents reproducible change with a high level of agreement and 2-step change is a Food and Drug Administration approved outcome in DR clinical trials.^19^^,^^20^ The proportion of ungradable image sets with each camera was documented. The image sets from each modality classified as ungradable (level 90) were excluded from the agreement tables for DRSS. All categorical variables are summarized as percentages. On all tests, *P* < 0.05 was considered significant, and nonparametric testing was applied where appropriate. All statistical tests were performed using R software (R version 4.2.2, R CoreTeam 2022, Vienna, Austria).

## Results

Fifty participants (N = 97 eyes) had 3 sets of color retinal images taken using standard 7F, Clarus, and Optos UWF-C imaging systems. Of these, the 7F region DR level was considered ungradable for 5 (5.2%) standard 7F image sets, 2 (2.1%) Clarus image sets, and 1 (1.0%) Optos image set. The global DR level was considered ungradable for 3 (3.1%) Clarus image sets and 3 (3.1%) Optos image sets. The distribution of subjects in each ETDRS category for standard 7F imaging, Clarus 7F region, Optos 7F region Clarus global, and Optos global is shown in Table 1.

**Table 1:** Distribution of Diabetic Retinopathy Severity Levels

ETDRS Steps	ETDRS Level	ICDR Severity	Standard 7-Field	Clarus 7-Field	Optos 7-Field	Clarus Global	Optos Global
			N = 97	N = 97	N = 97	N = 97	N = 97
1	10, 12, 14A-C, 15, 20	None/Early DR (4.1%)	4	7	10	4	3
2							
3	35A-F	Mild NPDR (48.5%)	47	35	37	30	35
4	43A-B	Moderate NPDR (20.6%)	18	20	22	21	25
5	47A-D		2	10	5	13	8
6	53A-E	Severe NPDR (2.0%)	2	3	2	3	2
7	60, 61A-B	Proliferative DR (19.6%)	7	8	9	8	10
8	65A-C		9	8	9	9	7
9	71A-D		3	4	2	6	4
10, 11, 12	75, 81, 85		0	0	0	0	0
90	90	Cannot Grade (5.2%)	5	2	1	3	3

DR = diabetic retinopathy; ICDR = International Clinical Diabetic Retinopathy; NPDR = non-proliferative diabetic retinopathy.

### Comparison of 7F ETDRS Levels Between the 3 Modalities

**Figure 2:**
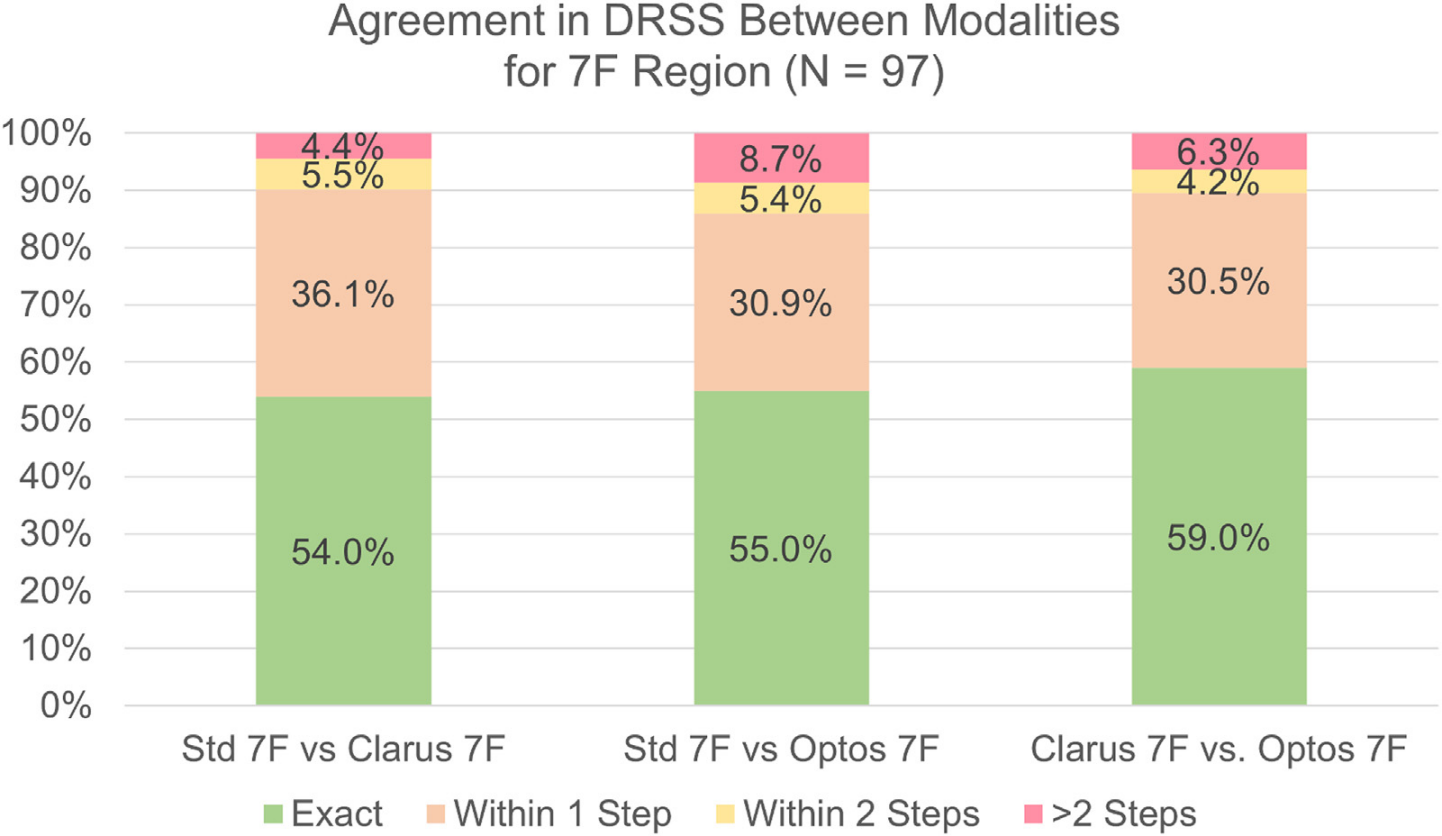
Stacked bar chart showing the exact agreement, 1-step agreement, 2-step agreement, and > 2-step agreement when assessing the Diabetic Retinopathy Severity Scale (DRSS) level in the 7-field (7F) area of standard 7F imaging, Clarus, and Optos.

The agreement rates for each of these modalities are shown in the stacked bar graphs in Figure 2. The DRSS 1-step agreement in the 7F region between standard 7F imaging and Clarus 7F was 90.1% (weighted κ [wκ], 0.65; 95% confidence interval [CI], 0.54–0.76). Comparing standard 7F imaging and Optos 7F, the 1-step agreement was 85.9% (wκ, 0.58; 95% CI, 0.45–0.71). Comparing the 2 UWF images, Clarus and Optos within the 7F region, the 2-step agreement was 89.5% (wκ, 0.68; 95% CI, 0.57–0.78). The detailed agreement on individual ETDRS levels between the modalities is shown in Fig S1A–C.

### Comparison of Standard 7F ETDRS Level to Global ETDRS Level with UWF Imaging

**Figure 3:**
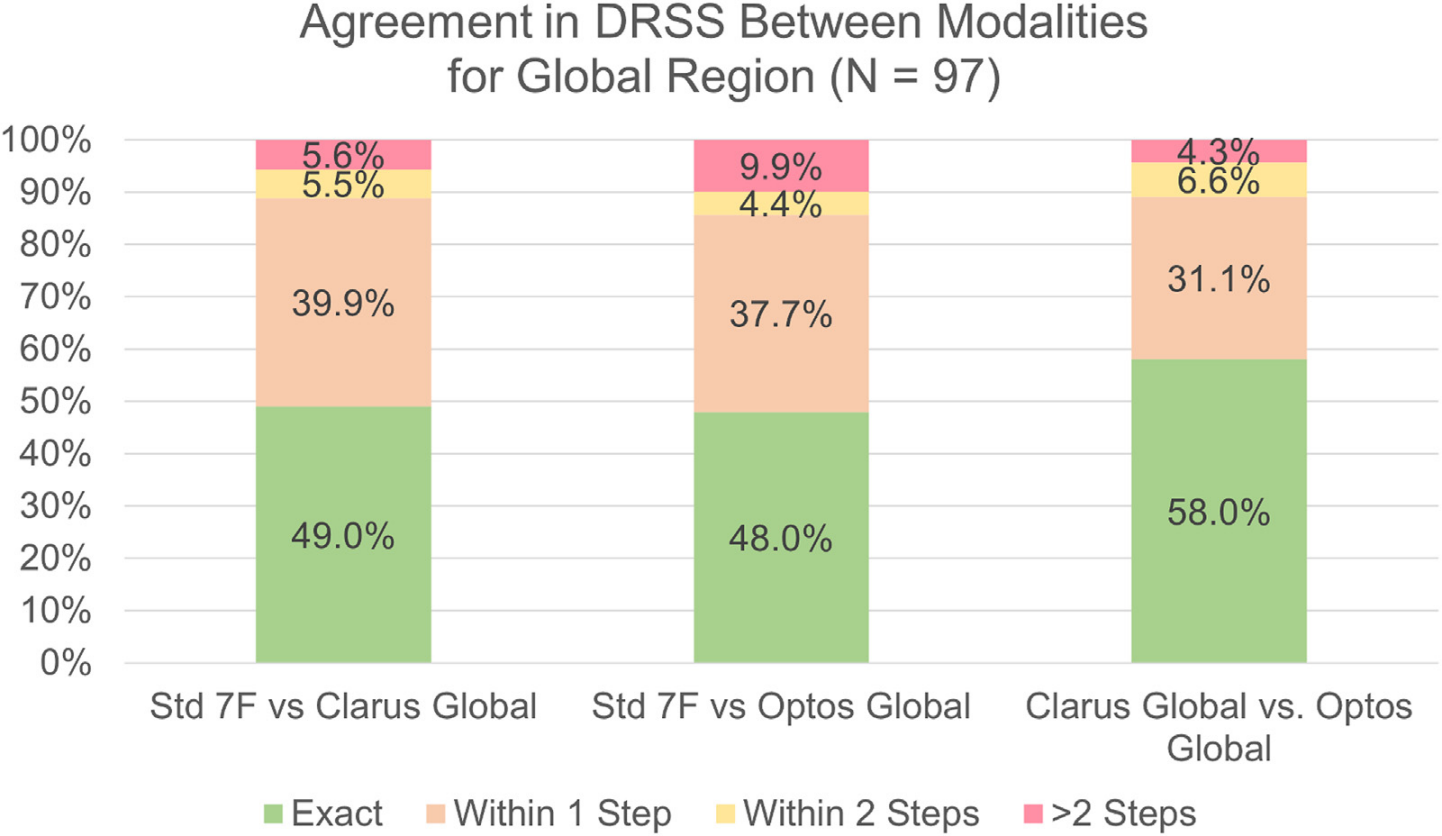
Stacked bar chart showing the exact agreement, 1-step agreement, 2-step agreement, and > 2-step agreement when assessing the Diabetic Retinopathy Severity Scale (DRSS) level in the 7-field (7F) area of standard 7F imaging and the global area of Clarus and Optos.

We explored if DRSS assessment beyond the 7F region affects the ETDRS level, i.e., the agreement between the standard 7F imaging ETDRS level and the global ETDRS level. The exact, 1-step, and 2-step agreement rates for standard 7F to global ETDRS levels are shown in the stacked bar graphs in Figure 3. The 1-step agreement between standard 7F imaging and UWF was 88.9% (wκ, 0.63; 95% CI, 0.51–0.74) for Clarus and 85.7% (wκ, 0.54; 95% CI, 0.41–0.67) for Optos. Clarus and Optos global levels were within 2 steps in 89.1% of the eyes (wκ, 0.68; 95% CI, 0.57–0.78). The agreement on each ETDRS level is shown in Fig S2A–C.

### Comparing 7F UWF ETDRS Level to Global ETDRS Levels

A comparison of the 7F ETDRS level and global ETDRS level within the same modality was also assessed. Within Clarus, the 7F and global levels agreed within 1-step in 96.9% (wk 0.89) and within Optos, 97.9% (wk 0.89).

### Disagreements Between NPDR and PDR Status

Disagreements in NPDR/PDR status between each modality and 7F/global are shown in Table 2. Overall, the numbers were small, ranging from 2 to 7, with no specific trend observed. Of 72 eyes graded as NPDR on standard 7F, 3 (4.1%) were graded as PDR on Clarus within the 7F and 5 (6.9%) on Optos. On the other hand, of 19 eyes graded as PDR on standard 7F, 2 (10.5%) were graded as NPDR on Clarus within 7F and 5 (2.6%) on Optos. In particular, within a UWF image, there were 3 (4.0%) eyes that changed from NPDR to PDR due to the inclusion of peripheral lesions with Clarus and 2 (2.7%) eyes with Optos.

**Table 2:** Discrepancies in Classification of Proliferatrive and Non-Proliferative Diabetic Retinopathy

	Proliferative Diabetic Retinopathy						
Non-proliferative diabetic retinopathy		Standard 7-Field	Clarus 7-Field	Optos 7-Field	Clarus Global	Optos Global	Total
	Standard 7-Field	N/A	3	5	4	6	72
	Clarus 7-Field	2	N/A	5	3	7	71
	Optos 7-Field	5	5	N/A	6	2	72
	Clarus Global	2	0	3	N/A	4	69
	Optos Global	5	5	0	5	N/A	72
	Total	19	20	19	21	19	

Intergrader agreement within each modality is shown in Table 3.

**Table 3:** Intergrader Reproducibility

Imaging Modality	Exact Agreement	1-Step Agreement	2-Step Agreement	wκ (SE)
Standard 7-Field	96%	96%	96%	0.84 (0.1)
Clarus 7-Field	85%	98%	98%	0.91 (0.03)
Clarus Global	89%	98%	98%	0.93 (0.03)
Optos 7-Field	76%	95.5%	96.4%	0.83 (0.04)
Optos Global	84%	97.3%	97.3%	0.89 (0.03)

SE = standard error; wκ = weighted kappa.

## Discussion

The ETDRS 7F imaging protocol has been the gold standard for assessing DR for the last several decades. Recent technological advances in fundus photography have included digital transition and field expansion from 30° retinal imaging, which has allowed for the development of 2 commercially available UWF imaging systems. In multicenter clinical trials utilizing different devices, it is important to establish comparability between instruments to pool the data for statistical analysis. In the COCO study, we compared the agreement in ETDRS level between 3 cameras: standard 7F imaging, relative to UWF-C systems Clarus and Optos. The agreement between all 3 devices was moderate to substantial for the full spectrum of DR levels. To be considered equivalent, a high agreement rate for DR level within 1-step is required between modalities as a 2-step agreement is the accepted outcome for clinical trials.^19^^,^^20^ One step agreement was > 85% and 2-step agreement between all 3 imaging techniques was > 95%, which supports the notion that both Clarus and Optos are equivalent to standard 7F imaging for obtaining 7F ETDRS levels in DR clinical trials.

There have been multiple studies comparing Optos to standard 7F imaging, but there is limited literature on the agreement of Clarus camera and Optos systems for the assessment of ETDRS levels. Hirano et al^21^ compared 50 paired Optos and Clarus images for assessment of 7F ETDRS level and found a κ agreement of 0.88. The UWF images were single shot on-axis and showed that the Optos field of view is nearly twice that of Clarus. Another study by Xia et al, compared all 3 modalities in a large cohort of Asian eyes. There was moderate agreement with standard 7F imaging and Optos (58.3% exact agreement, κ 0.45) compared to Clarus (93.8% exact agreement κ 0.92).^22^ While the Optos comparison to standard 7F imaging results in our study was similar at 56% exact agreement (κ 0.58), agreement with Clarus was not as high (55% exact agreement, wκ 0.65) and was comparable to Optos. Most of the disagreements in our study were for level 35 with both UWF images trending toward a higher level. A review of the disagreements found that the consensus group (N.D., N.B., and A.D.) agreed with the UWF grade in a majority of the eyes and attributed the lower grade in standard 7F imaging to poor image quality or due to a discrepancy in the field of view. As shown in Figure 1, a montage of the standard 7F images does not necessarily match the 7F region of the UWF images. Due to technician subjectivity, gaps exist between the various 30° fields where hemorrhages or intraretinal microvascular abnormalities (IRMA) can be missed. In addition, the effective gradable region on each 30° image is about 80% due to peripheral blurring and artifacts, further reducing the equivalent field of view. There were a few eyes at level 35 that were assigned a lower level with UWF imaging. In these eyes, the consensus group found that isolated round hemorrhages in a relatively large field of view in UWF images were being perceived as microaneurysms, resulting in a lower level.

**Figure 4:**
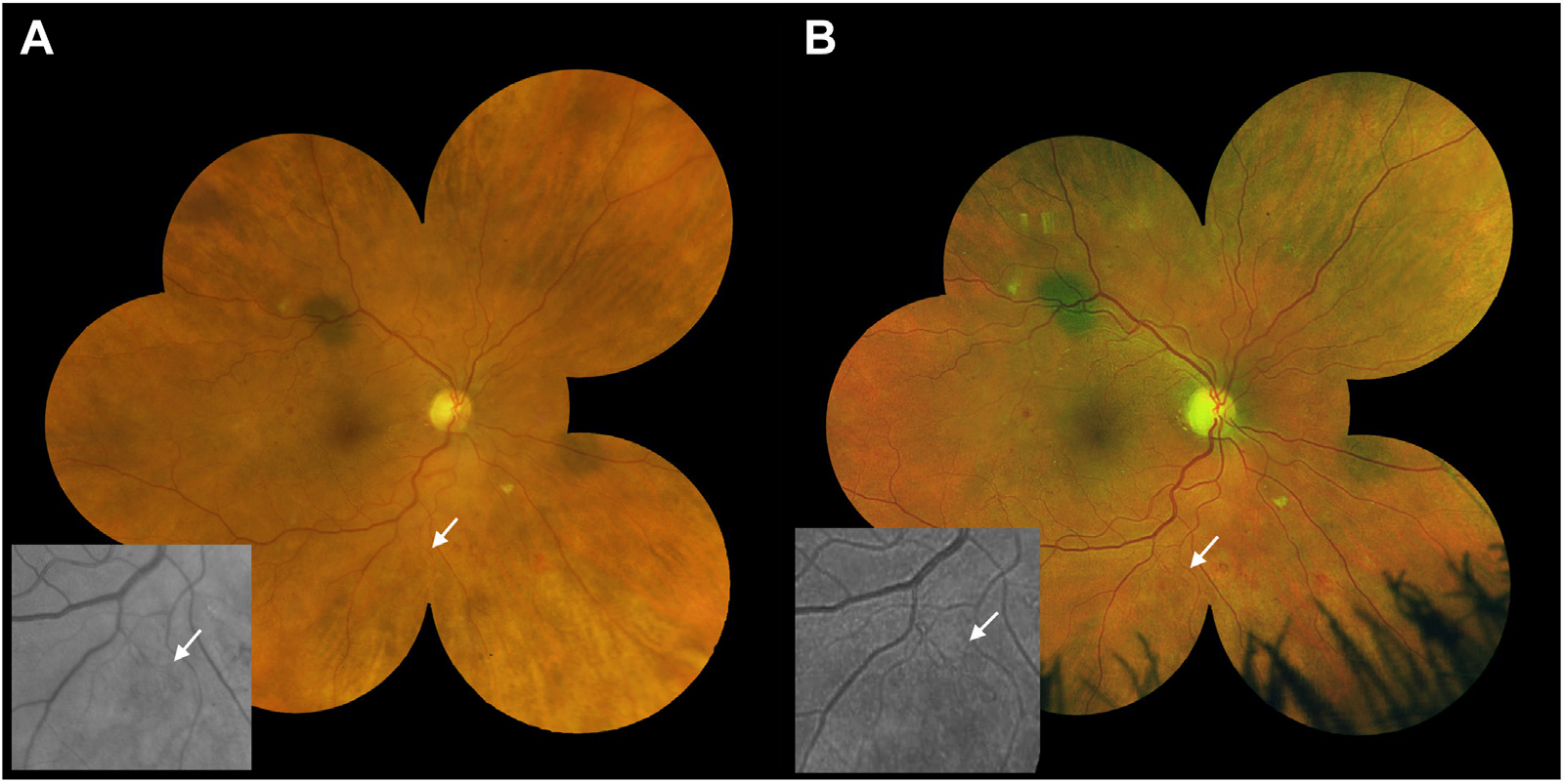
An example where new vessels were detected in the 7-field (7F) area of both Clarus (**A**) and Optos (**B**). This lesion was missed on standard 7F imaging due to poor field definition.

Although ETDRS level agreement is important for clinical trials, discrepancies in NPDR versus PDR levels are more significant clinically. The distinction between NPDR and PDR is clinically important as PDR is associated with a much higher risk of vision loss and requires more aggressive management. The discrepancy between NPDR/PDR occurred in both directions within every comparison pair. The first comparison is between the 7F region of all modalities. The shift ranged from 2% to 7% between each comparison pair and did not show any trend. Figure 4 shows an example where new vessels were not detected in standard 7F images due to poor field definition but were detected within the 7F area of Clarus and Optos.

**Figure 5:**
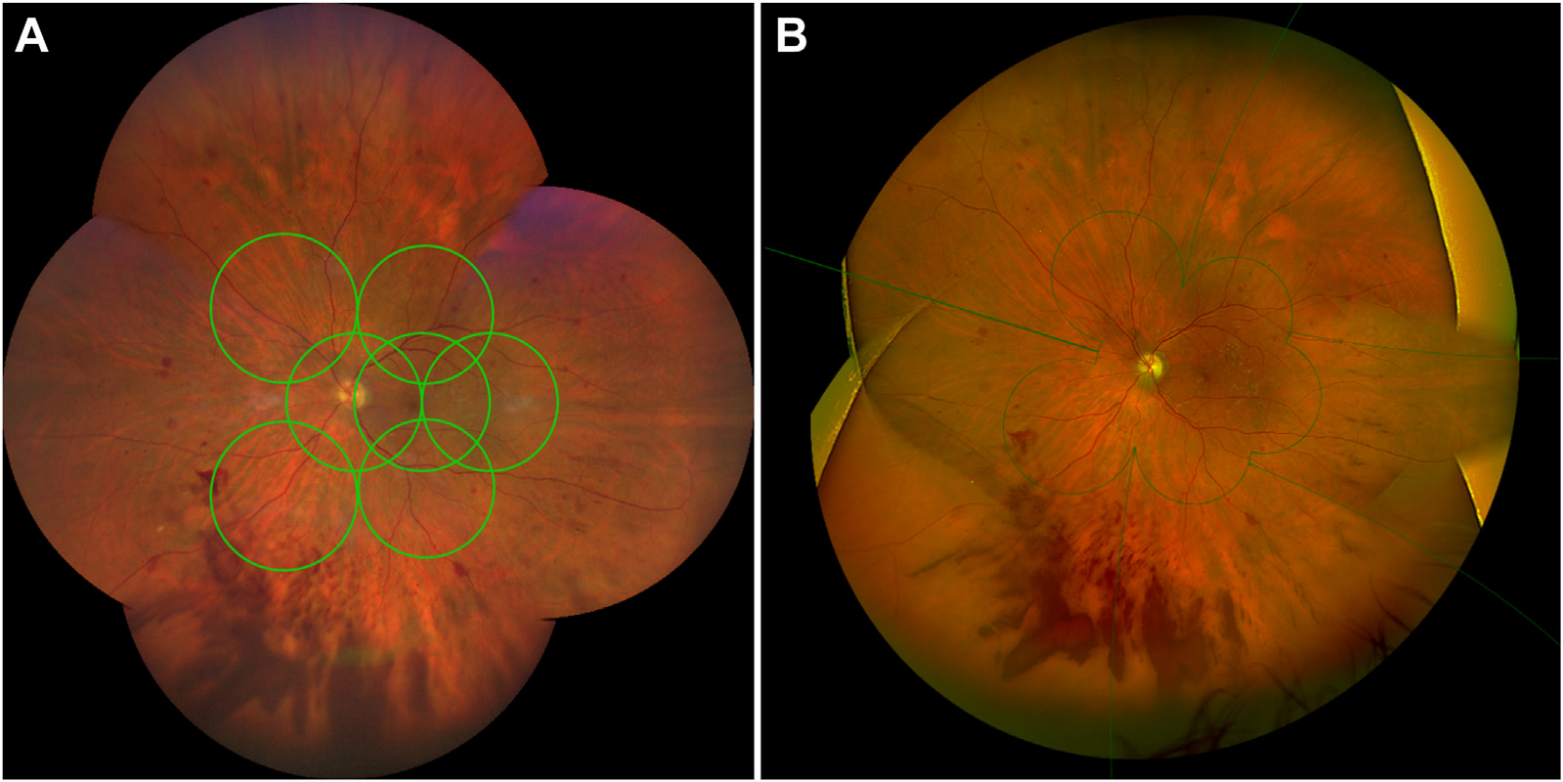
An example where there was a significant difference in Diabetic Retinopathy Severity Scale (DRSS) level between the 7-field and global areas within the same modality. The global DRSS was higher for both Clarus (**A**) and Optos (**B**) due to the presence of a large vitreous hemorrhage in the periphery.

A second area of interest is the occurrence of PDR outside the 7F region. Compared to standard 7F, PDR was seen in global DR assessment in 5.6% of Clarus images and 8.2% of Optos images. The effect of expanding beyond the 7F region on DRSS is best evaluated within a modality. We found a shift from NPDR to PDR status from 7F to global ETDRS levels in UWF imaging, with 4% of Clarus images and 2.7% of Optos images showing this change. Figure 5 shows an example where a large vitreous hemorrhage, mostly in the periphery, was detectable outside the 7F region on UWF images. This case contrasts with previous reports indicating that the region outside the 7F does not significantly affect the DRSS.^7^^,^^11^^,^^14^^,^^23^ Interestingly, there were images (4% for both Clarus and Optos) that were classified as NPDR on UWF and PDR on standard 7F imaging. In most of these eyes, small new vessels were seen on standard 7F imaging but went undetected on Clarus and Optos due to image quality issues such as focus and saturation.

Another comparison is between global levels of Clarus and Optos, which are expected to be similar, particularly with the steered imaging and montaging that creates an equivalent field of view. However, we observed an equal distribution of eyes, approximately 6% to 7%, that exhibited an NPDR/PDR shift between the 2 modalities, with no particular modality consistently selected for consensus. This indicates that, despite the advantages of 3-color channels and improved resolution with Clarus, DR assessment can still vary to a small extent.

### Ungradable Images

The proportion of ungradable images was higher in standard 7F imaging (5.2%) compared with the 7F region for Clarus (2.1%) and Optos (1.0%). Common reasons for an ungradable image set in the standard 7F imaging group included technical issues, such as missing images, or poor camera focus obscuring ≥ 2 fields by ≥ 50%. There were also patient-related issues, such as a dense lens opacity or secondary cataract compromising image quality. When considering the global region, there was an equal number of ungradable images for both Clarus (3.1%) and Optos (3.1%). This contrasts with our previous publication where the Optos images were ungradable in nearly 25% of the data.^14^ However, the images in that study were obtained from 2019 to 2020 and increased experience with the camera could account for the difference. In addition, the previous study was multicentered, accounting for various levels of experience in a more advanced disease population compared to the current single-center study.

### Grader Reproducibility

A change by ≥ 2 steps in the DRSS scale is a clinically significant outcome in DR clinical trials.^19^^,^^20^ By this standard, the agreement between graders was excellent for standard 7F imaging, Clarus, and Optos, as it was > 95% for 1-step and 2-step agreement in all modalities. The exact agreement for the 7F ETDRS level between the 2 graders within each modality is 76% with Optos, 85% with Clarus, and 96% with standard 7F imaging. The exact agreement is higher than historical data for standard 7F imaging from the Wisconsin Reading Center, which has been around 50% to 70%.^14^^,^^17^^,^^24^ The high agreement is possibly due to good-quality images taken from a single site with cooperative participants. In addition, ETDRS levels 43 to 53 (moderate and severe NPDR) are some of the most difficult to grade consistently and can affect reproducibility. In our dataset, only 22% of the images accounted for these levels, which could have resulted in higher reproducibility.

### Strengths and Limitations

The COCO study obtained high-quality digital fundus photographs from each patient, captured during a single visit by certified photographers. A notable strength is the detailed evaluations of DRSS for each image set, carried out by experienced graders in a reading center. Montaging the images creates a comparable field of imaging for both UWF modalities. Despite a skewed stratification with 48% of eyes in the mild NPDR group, we were able to compare the 3 modalities across the full spectrum of DR severity, including various PDR levels. Evaluation of the global DRSS for each UWF camera is also a unique element of this study. While there are multiple comparisons in the literature between a single UWF imaging system and standard 7F imaging, head-to-head comparison of all 3 devices is limited. Demonstrating agreement between cameras using the ETDRS scale, rather than the International Clinical Diabetic Retinopathy scale, is necessary for future use in clinical trials. Limitations of this study include that it was performed at a single-center retina practice, whereas the involvement of multiple clinical centers could have allowed for an even larger sample size and enhanced generalizability.

## Conclusion

In conclusion, the ETDRS level within the 7F area can be accurately established with either Clarus or Optos imaging systems and is comparable to the long-established method of standard 7F imaging. Additionally, the global DR severity level can be determined equally well with either Clarus or Optos. In a few cases, employing the UWF-C cameras to visualize outside the 7F region allowed proliferative lesions to be detected, which raised the DR severity level. If a facility has access to multiple types of imaging systems, switching between different devices could potentially result in a patient’s DRSS level changing due to variations in the imaging system; this could affect the accuracy of monitoring disease progression. Therefore, it is necessary for ophthalmologists to be aware of the relative features of each camera to ensure reliable tracking of a patient's DR severity.
