# Supplementary material for: Comparison of Standard 7-Field, Clarus, and Optos Ultrawidefield Imaging Systems for Diabetic Retinopathy (COCO Study)
Source: Ophthalmol Sci. 2023 Nov 11;4(3):100427. doi: 10.1016/j.xops.2023.100427 (PMC10818251; doi:10.1016/j.xops.2023.100427)

Supplemental Material

FIGURE 1. Cross tables illustrating the agreement at each ETDRS level for the 7-field area between standard 7-field imaging and Clarus (1a), standard 7-field imaging and Optos (1b), and Clarus and Optos (1c).

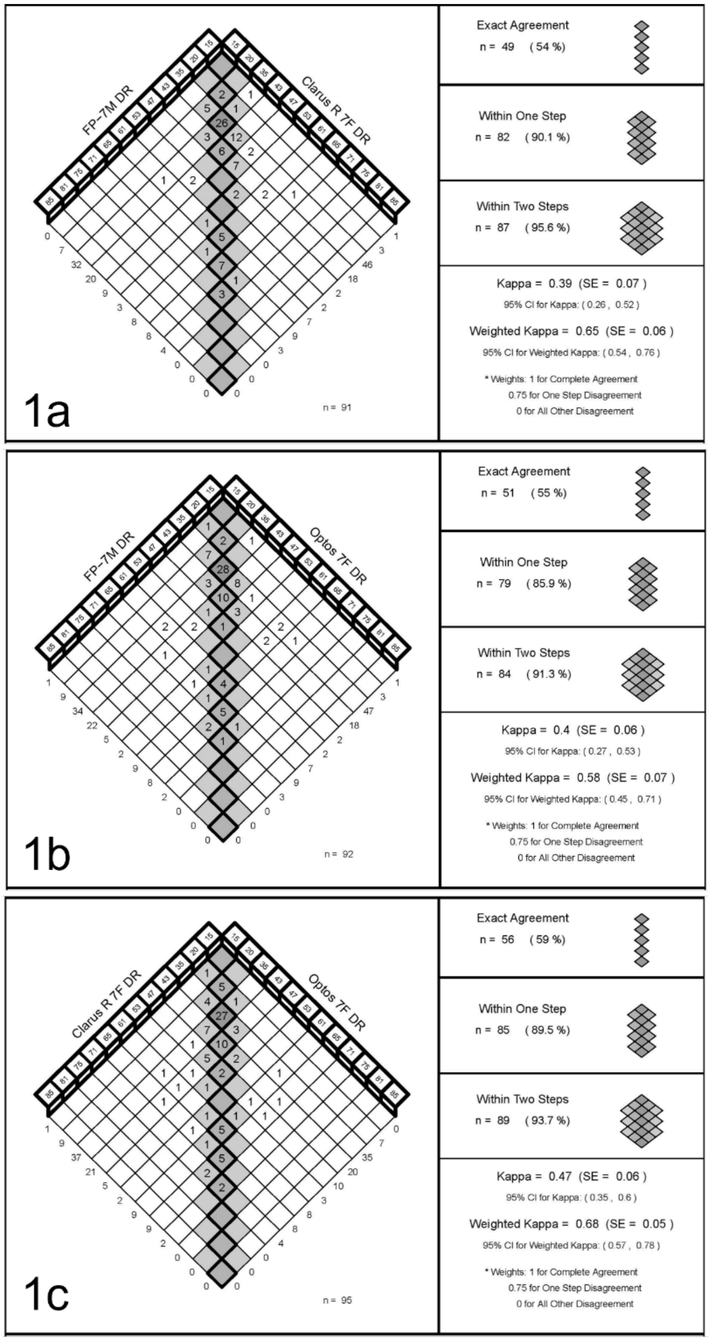

FIGURE 2. Cross tables illustrating the agreement at each ETDRS level between standard 7-field imaging and the global DRSS with Clarus (2a), and standard 7-field imaging versus the global DRSS with Optos (2b). The agreement between both ultra-widefield modalities was also assessed (2c).

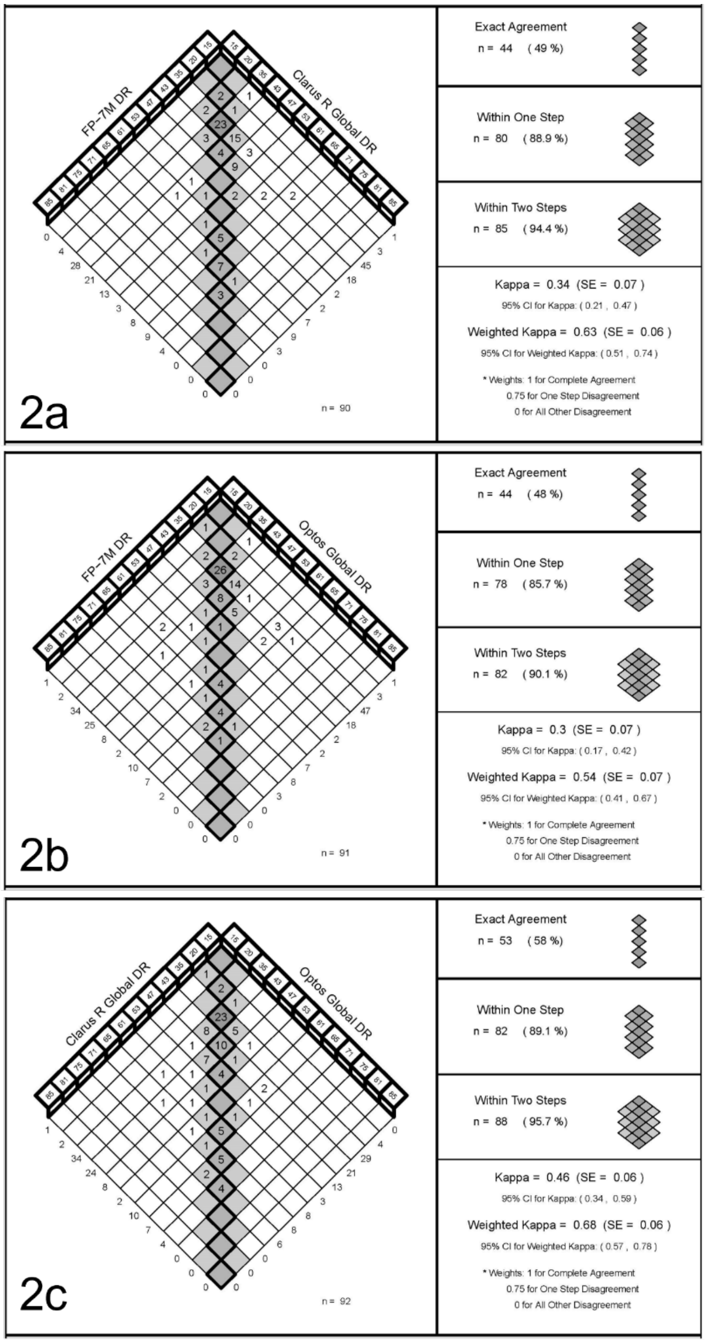

FIGURE 3. One of many examples where the standard 7-field imaging (A) was categorized as mild non-proliferative diabetic retinopathy (NPDR), but both Clarus (B) and Optos (C) were categorized as moderate NPDR due to the detection of additional hemorrhages, and/or intraretinal microvascular abnormalities (IRMA).

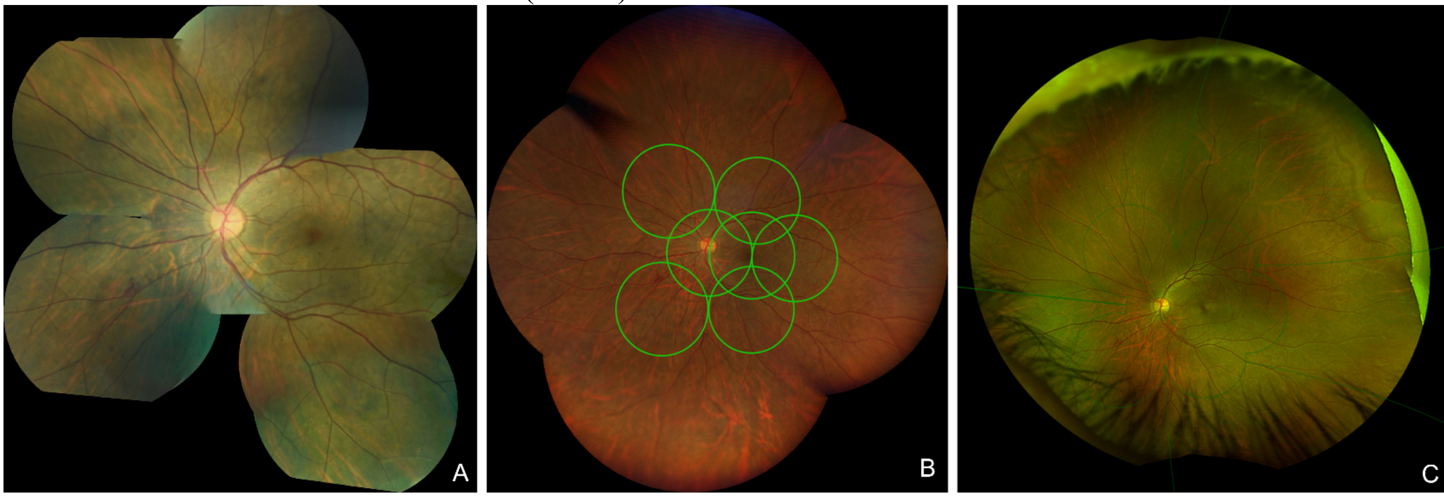

Supplement: Supplemental Figures [file mmc1.pdf]
